# Multimodal Imaging of the Corneal Endothelial Transition Zone Reveals Progenitor Cell Population

**DOI:** 10.3390/cells14231851

**Published:** 2025-11-25

**Authors:** Sonika Rathi, Patricia Hülse, Susanne Staehlke, Marcus Walckling, Mahmoud Anwar, Peter Trosan, Sebastian Bohn, Oliver Stachs, Gary S. L. Peh, Gary Hin-Fai Yam, Jodhbir S. Mehta, Nicola Hofmann, Martin Börgel, Thomas A. Fuchsluger

**Affiliations:** 1 Department of Ophthalmology, Rostock University Medical Center, 18057 Rostock, Germany; 2 Institute of Cell Biology, Rostock University Medical Center, 18057 Rostock, Germany; 3 Department of Life, Light & Matter, Interdisciplinary Faculty, University of Rostock, 18059 Rostock, Germany; 4 Regenerative Therapy Group, Singapore Eye Research Institute, Singapore 169856, Singapore; 5 Corneal Regeneration Laboratory, University of Pittsburgh School of Medicine, Pittsburgh, PA 15219, USA; 6 Corneal & External Eye Disease, Cataract & Comprehensive Ophthalmology, Refractive Surgery, Singapore National Eye Centre, Singapore 168751, Singapore; 7 German Society for Tissue Transplantation (DGFG), 30625 Hannover, Germany

**Keywords:** corneal endothelium, transition zone, endothelial progenitor cells, stem cell markers, confocal microscopy

## Abstract

**Highlights:**

**What are the main findings?**

- Central endothelial cell (EC) density decreases with donor age and storage time, but the transition zone (TZ) remains structurally and quantitatively stable.
- The TZ shows distinct morphology and expression of progenitor markers, indicating stem cell–like regenerative potential.

**What are the implications of the main findings?**

- TZ may play a role in corneal endothelial regeneration and could therefore be considered in further donor cornea assessments.
- Non-invasive imaging (HRTII/RCM) could enable better evaluation of TZ health and may improve donor cornea selection in the future.

**Abstract:**

The assessment of donor corneas is currently based solely on central endothelial cell (EC) density, which potentially overlooks the transition zone (TZ) regenerative potential. Therefore, the present study characterizes TZ using multimodal imaging techniques to understand its regenerative potential and refine the assessment of donor tissue. Ex vivo donor corneas (n = 41) were examined using phase-contrast microscopy for EC counting and reflectance confocal microscopy (HRTII/RCM) for non-invasive visualization of the TZ. A subset of eight of these corneas underwent ultrastructural analysis using field-emission scanning electron microscopy (SEM) and immunostaining analysis using confocal microscopy. We observed a significant decrease in central EC density (*p* < 0.001) with increasing storage duration and donor age, while TZ width and TZ surface cell count remained stable. HRTII/RCM and SEM revealed distinct morphological differences (small, polygonal cells, irregular arrangement) in the TZ compared to the peripheral endothelium (PE). Immunostaining revealed elevated expression of progenitor markers (Nestin, ABCG2, SOX2, Lgr5, Vimentin) and reduced expression of endothelial markers (ZO1 and Na/K-ATPase) in the TZ compared to the PE, indicating the presence of a stem cell-like population. These findings suggest that TZ may contribute to endothelial cell regeneration, and HRTII/RCM could serve as a novel tool for TZ evaluation in low EC count donor corneas.

## 1. Introduction

The corneal endothelium plays a crucial role in maintaining corneal transparency by regulating fluid balance. However, aging, diseases such as endothelial dystrophies, glaucoma, surgical trauma, and injury can cause corneal endothelial cell (EC) loss, which leads to endothelial dysfunction and corneal decompensation that impairs vision [1]. Cadaveric donor cornea transplantation is the standard treatment to restore endothelial function in such cases. Usually, evaluation of donor corneas for transplantation is primarily based on central EC density, with a threshold of >2000 cells/mm [2,3]. This often leads to the rejection of donor tissue with a lower EC density (<2000 cells/mm^2^), while the endothelial transition zone (TZ) of the cornea is often neglected. However, recent evidence suggests that the TZ may harbor progenitor cells with regenerative potential that could influence corneal health and repair mechanisms [4,5].

**Figure 1:**
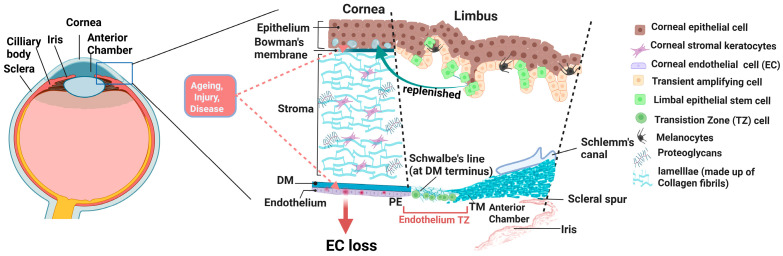
Schematic representation of the human anterior segment highlighting the cornea, limbus, endothelial transition zone, and trabecular meshwork regions. The figure depicts the anterior segment, including cornea, limbus, TZ, and TM, along with other anterior chamber structures. The corneal architecture includes epithelium, Bowman’s layer, stroma, Descemet’s membrane, and the endothelium. The corneal endothelium is in continuity with the endothelium TZ at the termination of DM, which harbors progenitor-like cells (green, round). Transient amplifying cells (beige) and limbal epithelial stem cells (green, square) present in the anterior limbus continuously renew corneal epithelial cells (green, square), while the endothelium has post-mitotic cells that, when affected by age, trauma, or diseases, lead to endothelial cell loss that can impair corneal integrity and function (adapted from Xiao Y et al. [10] and created with BioRender.com, agreement No. ED28KL4L0C). EC: Endothelial Cell, TZ: Transition zone, PE: Peripheral endothelium, TM: Trabecular meshwork, DM: Descemet’s membrane.

The cornea structurally consists of five layers: the epithelium, Bowman’s layer, stroma, Descemet’s membrane, and endothelium (Figure 1) [6]. The stroma is the largest part of the cornea, providing mechanical strength and transparency with its structured collagen fibers and proteoglycans (Figure 1) [6,7]. The endothelium, which consists of hexagonal cells with tight junctions arranged in a tessellated form, maintains the hydration and transparency of the stroma [1]. Unlike the corneal epithelium, EC does not regenerate in vivo due to cell cycle arrest and the presence of growth inhibitory factors like transforming growth factor-beta (TGF-β) in the aqueous humor [8,9]. This limitation makes the endothelium vulnerable to aging, disease, and injury, which can gradually cause a decline in endothelial cells (Figure 1).

The TZ, also referred to as the smooth or flat zone, is located at the posterior limbus and marks the boundary between the corneal endothelium and the trabecular meshwork (TM) (Figure 1) [11]. Notably, both the corneal endothelium and the TM originate from the neural crest and have limited proliferation capacity, but have distinct functional properties, with the EC involved in corneal hydration and TM cells maintaining aqueous humor outflow [12,13]. Studies in human and animal corneas suggest that the TZ contains stem/progenitor cells capable of repopulating both the corneal endothelium and the TM [4,5]. The evidence of repopulation of the central endothelium in Fuchs’ Endothelial Corneal Dystrophy (FECD) patients, either in the absence or after detachment of the donor graft [14,15], indicates the presence of endogenous progenitor cells that might be capable of corneal endothelial repair. Similarly, in some cases of glaucoma, after treatment with argon laser trabeculoplasty, the proliferation of cells was observed in the TM, which further supports the hypothesis that progenitor cells may migrate and repopulate the endothelial cells in the TM [16]. This regenerative potential aligns with recent studies identifying the existence of stem or progenitor cells in the TZ, which could contribute to endothelial regeneration [4,17]. Within the posterior limbus, Schwalbe’s line (SL) is a curved ridge at the anterior chamber angle that marks the termination of Descemet’s membrane and the beginning of the non-filtering part of the TM (Figure 1), which has also been reported to harbor stem/progenitor cells with both TM and endothelial regenerative potential [18]. Also, the presence of markers for embryonic stem cells (like Sox2, Oct4, Nanog), neural crest stem (Nestin, p75NTR, Sox9), and mesenchymal markers in TZ and SL [17,19] suggests that the peripheral endothelium (PE) and TM likely share the same stem cell origin. Isolation and ex vivo differentiation of cells from TZ into endothelial cells with expression of ZO1, Na/K-ATPase marker [17], and TM cells with the expression of TM markers like TIMP3, AQP1, and CHI3L [19] further establishes that TZ cells contain the stem cell population for both corneal endothelium and TM regeneration.

Based on previous findings, TZ represents a promising niche for endothelial regeneration in both the cornea and TM. In this study, we employed advanced imaging techniques to characterize the regenerative potential of the TZ to improve, with the aim of improving donor cornea assessment. Specifically, we analyzed the influence of donor age and storage time on endothelial and progenitor cell density and spatially localized the progenitor cell populations within the TZ. For this purpose, we used phase contrast microscopy, reflectance confocal microscopy, scanning electron microscopy, and laser scanning confocal microscopy to obtain high-resolution insights into the ultrastructural organization of the TZ. A comprehensive evaluation of this region may help reduce donor tissue discard rates and facilitate the development of new therapeutic strategies for stimulating endogenous endothelial repair before transplantation, particularly in borderline corneas where identical endothelial cell densities (e.g., in 2400 cells/mm^2^) differ in suitability depending on transition zone quality.

## 2. Materials and Methods

### 2.1. Ex Vivo Human Donor Corneas

In this study, 41 donor corneas, deemed unsuitable for transplantation or retrieved post-transplantation, were obtained from the Cornea Bank DGFG (Deutsche Gesellschaft für Gewebetransplantation, Network for Tissue Donation, Tissue Processing, and Transplantation, Hannover and Rostock, Germany). All research involving human tissue was approved by the local ethics committee and conducted in accordance with the Declaration of Helsinki (Ethics approval number: A 2020-0108). Informed consent was obtained either from the donors during their lifetime or from the donors’ next of kin. Before examination of the donor tissue, the following parameters were recorded: donor age (in years), gender (male/female), and total preservation time prior to grafting/examination (in days). Corneas were stored in culture medium I (P04-09701, PAN Biotech GmbH, Aidenbach, Germany) at 37 °C, then transferred to dextran-supplemented culture medium II (P04-09702, PAN Biotech GmbH, Aidenbach, Germany) at room temperature (RT), up to a maximum of six days before the experiment, to reverse swelling.

### 2.2. Phase Contrast Microscopy

Phase-contrast microscopy was used to assess the central corneal EC density in donor corneas. High-quality images were obtained from 41 donor corneas before examination/transplantation utilizing a Leitz phase-contrast microscopy system (Leitz Leica Labovert FS, Wetzlar, Germany). For each cornea, three digital images were captured and evaluated. Central endothelial cell counts were manually performed within a 0.02 mm^2^ frame by two experienced examiners, and the average of three images was used to determine the final cell density to ensure accuracy and consistency.

### 2.3. Reflectance Confocal Microscopy with Intrinsic Contrast Using Heidelberg Retina Tomograph Equipped with the Rostock Cornea Module (HRTII/RCM)

The corneas were divided into four equal segments, each analyzed individually using reflectance confocal microscopy with intrinsic contrast (a modified Heidelberg Retina Tomograph equipped with the Rostock Cornea Module, Heidelberg Engineering GmbH, Heidelberg, Germany), as described previously [20]. This is a non-invasive imaging technique that generates transversal images (enface images), with high resolution and excellent depth discrimination. For imaging, a disposable contact cap (TomoCap^®^, Heidelberg Engineering GmbH, Heidelberg, Germany) was optically coupled to the microscope lens (Achroplan 63×/W, NA 0.95; Carl Zeiss, Jena, Germany) and the cornea using an aqueous tear gel (Vidisic^®^, Bausch & Lomb/Dr. Gerhard Mann, Berlin, Germany). Each corneal segment was scanned in at least four distinct corneal TZ areas to ensure comprehensive coverage. The HRTII/RCM is one of the established confocal in vivo imaging systems in ophthalmology. Basically, the HRT imaging device has a 670 nm laser diode originally developed for analyzing the optic nerve head to detect glaucomatous damage. The ‘Rostock Cornea Module’ (RCM) converts the HRTII into a high-resolution confocal laser scanning microscope for visualizing the anterior segment of the eye [21,22]. The HRTII/RCM scans captured an area of 400 × 400 µm (384 × 384 pixels per image), with a transversal optical resolution of 1–2 µm and longitudinal optical resolution of 4 µm. The captured images were analyzed to determine TZ width and cell density in the donor corneas with ImageJ (Version 2.1.0/1.53c). Cell counts were performed manually within a 100 µm^2^ frame in each of the four sections. Furthermore, TZ width was measured as the linear distance between TM and PE such that the inner border of TZ is adjacent to the PE, and the outer border is demarcated by the anterior non-filtering TM beam inserts and bridges.

### 2.4. Scanning Electron Microscopy

The TZ in human donor corneas was analyzed ultrastructurally using a Field Emission Scanning Electron Microscope (SEM; Merlin VP Compact, Carl Zeiss AG, Oberkochen, Germany). For SEM analysis, the corneas were subdivided into four equal parts and fixed in fixative solution containing 1% paraformaldehyde (PFA, Merck KGaA, Darmstadt, Germany), 2% glutaraldehyde (GA, Merck KGaA), and 0.1 M sodium phosphate buffer (Na-P, Merck KGaA) at 4 °C. These fixed samples were washed in 0.1 M Na-P buffer, post-fixed in 1% osmium tetroxide (OsO3, Thermo Fisher Scientific Inc., Darmstadt, Germany), and further washed in distilled water followed by dehydration in 30%, 50%, 70%, and 90% ethanol and twice in absolute ethanol. After these procedures, samples underwent Emitech 850 critical point drying (Quorum Technologies Ltd., Laughton, East Sussex, UK) to minimize distortion to the sample. To increase the conductivity of the samples for high-resolution imaging, the samples were sputter-coated with 10 nm of gold using EM SCD 004 (BAL-TEC AG, Balzers, Liechtenstein). The corneal sections were observed using SEM (acceleration voltage 5 kV and working distance of 6–14 mm). Imaging was performed at magnifications ranging from 25× to 50,000× to obtain high-resolution views of TZ, TM, PE, and other corneal structures.

### 2.5. Whole Mount Immunohistochemistry

Donor corneas were immunohistochemically analyzed to evaluate the TZ, PE, and TM using antibodies targeting endothelial markers (ZO1 and Na/K-ATPase) and stem/progenitor cell markers (Nestin, Lgr5, ABCG2, Vimentin, and SOX2). Corneas were first quartered and fixed in 2% PFA at RT for one hour, followed by rinsing with PBS and permeabilization in 0.1% Triton X-100 for 30 min at RT. Samples were then blocked with 2% bovine serum albumin (BSA) and 5% fetal calf serum for 1 h at RT, and incubated with primary antibodies (Table S2) overnight at 4 °C. After washing, samples were incubated with fluorophore-conjugated secondary antibodies (Table S2) in the dark at RT. Finally, samples were washed and mounted in Fluoroshield containing DAPI (Sigma-Aldrich, St. Louis, MO, USA). Immunostained corneal tissues were visualized using an inverted confocal laser scanning microscope (cLSM780, Carl Zeiss AG, Oberkochen, Germany). High-resolution Z-stack images (1 µm thickness) were acquired with a 10× objective using the cLSM780, and three-dimensional reconstructions were generated using ZEN software (Version ZEN 2011 SP4, Black Edition, Carl Zeiss, Germany). Fluorescence intensity was quantified using ImageJ (Version 2.1.0/1.53c) to calculate corrected total cell fluorescence (CTCF).

### 2.6. Statistical Analysis

Statistical analysis was performed using the averages of EC density, TZ cell count, and TZ width with GraphPad Prism software (Version 9, GraphPad Software, San Diego, CA, USA). Quantities were graphed against storage time and age groups and presented as mean ± standard deviation (SD). Linear regression analysis was performed to analyze the correlation between donor age and EC density. Data representing the effect of age and organ storage duration on EC density, TZ width, and TZ cell count were quantified using either a parametric *t*-test (if comparing two groups) or one-way ANOVA (if comparing more than two groups). Post hoc analyses were performed using Tukey’s multiple comparisons test, with statistical significance set at *p* < 0.05.

## 3. Results

### 3.1. Human Donor Characteristics and Corneal Endothelial Cell Density Distribution

The study analyzed 41 donor corneas (mean age: 71.6 ± 10.4 years, range: 45 to 87 years), which had been stored between 15 and 35 days. Of the donors, 32 were male and 9 were female. Corneal endothelial cells were visualized as hexagonal, mosaic-like structures in donor corneas using phase-contrast microscopy (Figure S1), and images were captured to determine the average endothelial cell (EC) density. The average central EC count was 2382.80 ± 405.64 cells/mm^2^, and the corneal TZ cell count was 2107.81 ± 505.03 cells/mm^2^ (Table 1). The organ storage period for the female donor corneas was 15–28 days; therefore, eight male donor corneas stored for more than 28 days were excluded from the gender comparison. The age distribution between male (71.2 ± 10.2 years old, n = 24) and female (73.0 ± 12.4 years old, n = 9) donors was comparable (Table S1). A decrease in central EC density with advancing age was observed in both the male (*p* = 0.023, n = 24) and female (*p* = 0.04, n = 9) groups. Still, no significant difference in EC distribution was observed between the genders (Table S1 and Figure S2). Due to the limited number of female donors, definitive sex-based differences in EC density could not be concluded.

### 3.2. Imaging Modified Reflectance Microscopy

**Figure 2:**
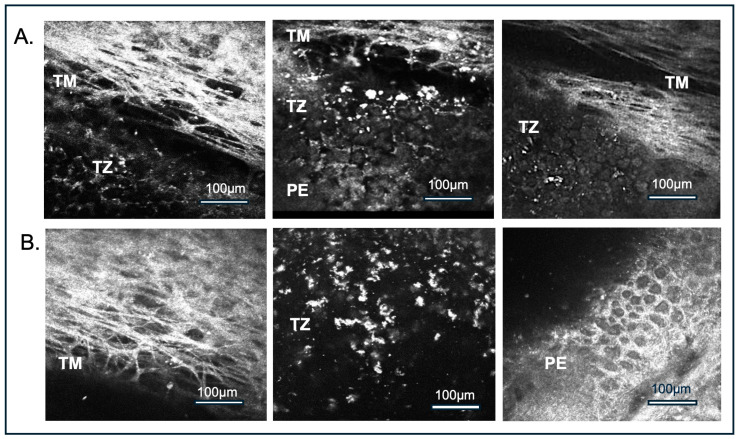
Representative ex vivo corneal confocal microscopy (HRTII/RCM) images of the corneal endothelial transition zone. Panel (**A**). Sequential imaging of the trabecular meshwork (TM), transition zone (TZ), and peripheral endothelium (PE); (**B**). The peripheral region displays fibrous structures of the TM, followed by a punctate dot-like pattern representing cells with irregular morphology and irregular arrangement in TZ, as compared to PE with tightly packed hexagonal morphology. Scale bar: 100 µm.

Ex vivo corneal confocal imaging was performed on all 41 donor corneas using the HRTII/RCM, showing the anatomical transition from the TM to TZ and PE (Figure 2). This imaging system clearly delineates the TZ, which appears as a distinct, blank region interspersed with irregular white punctate structures between TM and PE. These punctate structures likely correspond to cellular elements within the TZ and are referred to as TZ cells in the present study (Figure 2), which was validated by SEM and immunostaining with DAPI and stem cell markers demonstrating DAPI—positive nuclei and cellular morphology in this region.

On the surface facing the anterior chamber, TZ cells displayed heterogeneous sizes and were interspersed with gaps, unlike the adjacent PE, which exhibited a regular mosaic of tightly packed, hexagonal cells (Figure 2).

Measurements of the TZ width and TZ surface cell count were conducted by HRTII/RCM. The mean TZ width was 221.1 ± 40.9 µm, and the average TZ cell surface density was 2107.81 ± 505.03 cells/mm^2^ (Table 1), which was just slightly lower (10–12%) than the central EC density. The TZ width was comparable between male and female donors, although individual variability was observed. A slightly higher TZ cell density was noted in female donors compared to males; however, this difference was not statistically significant, likely due to our cohort’s limited number of female donor corneas in our cohort (Table S1).

### 3.3. Influence of Storage Time on Corneal Endothelial Cell Density, Transition Zone Width, and Cell Count

**Figure 3:**
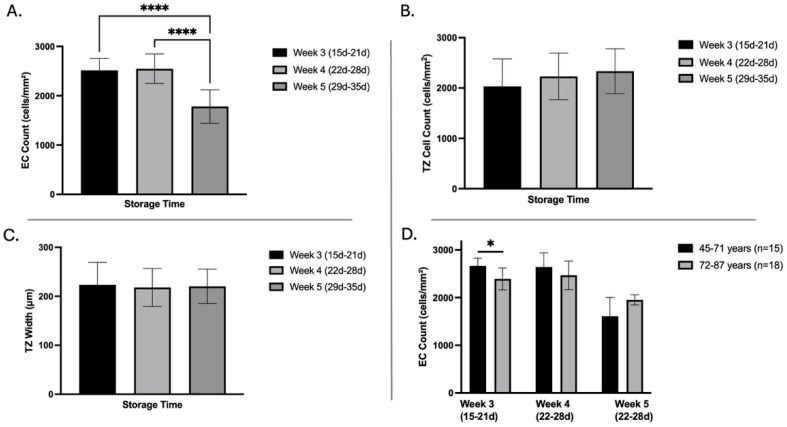
Effect of storage time on donor corneas. (**A**): endothelial cell (EC) count; (**B**): transition zone (TZ) cell count; (**C**): TZ width; (**D**): EC count in age groups with different organ storage duration. Bar graphs represent the mean ± standard deviation. Statistical significance is indicated by **** (*p* < 0.001) and * (*p* < 0.05).

Central EC density declined significantly with prolonged organ storage: after three weeks (15–21 days, n = 20), the mean EC density was 2516 ± 241 cells/mm^2^; it remained stable at four weeks (22–28 days, n = 13, 2548 ± 299 cells/mm^2^; week 3 vs. week 4: *p* = 0.9). By the fifth week (29–35 days, n = 8), EC density dropped significantly to 1781 ± 338 cells/mm^2^ (week 3 vs. week 5 and week 4 vs. 5: *p* < 0.001) (Figure 3A). In contrast, the number of TZ surface cells, as assessed by HRTII/RCM, showed no significant change over time (week 3: 2032 ± 546 cells/mm^2^, week 4: 2230 ± 464 cells/mm^2^, week 5: 2334 ± 446 cells/mm^2^, see Figure 3B,C). Similarly, TZ width remained stable across all storage durations (week 3: 224 ± 46 µm, week 4: 218 ± 39 µm, week 5: 220 ± 35 µm). Donor age, in combination with organ storage time, was associated with a significant decrease in EC count in corneas from older donors (72–87 years, 2665.6 ± 162.95 cells/mm^2^; n = 9) compared to those from younger donors (45–71 years, 2393.6 ± 229.1 cells/mm^2^; n = 11, *p* = 0.008, Figure 3D). By the fifth week of storage, EC density declined significantly compared to the third and fourth weeks, independent of donor age (week 3 vs. week 5: *p* = 0.059; week 4 vs. week 5: *p* = 0.054, Figure 3D). The impact of prolonged storage on EC density was so significant that it masked any potential age-related differences in EC loss.

### 3.4. Influence of Age on EC Density, TZ Width, and Cell Count

**Figure 4:**
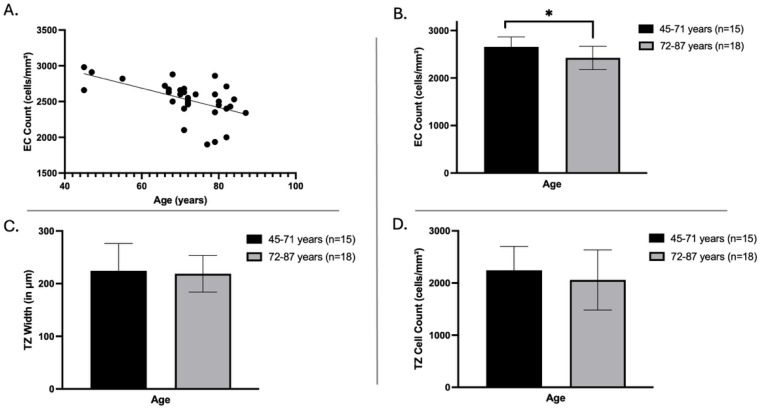
Effect of age on donor corneas. (**A**): endothelial cell (EC) count with increase in age; (**B**): EC count in different age groups; (**C**): transition zone (TZ) width; (**D**): TZ cell count in different age groups. Bar graphs represent the mean ± standard deviation. Statistical significance is indicated by * (*p* < 0.05).

An analysis was conducted to understand the effect of age on EC density in donor corneas, excluding eight corneas stored for 29–35 days (week 5) to avoid the masking effect of prolonged storage, with the remaining corneas having a mean storage time of 21 days. A significant inverse correlation between donor age and EC density (*p* = 0.0009, n = 33) (Figure 4A) was observed by linear regression analysis.

To further understand the effect of age on EC density, the data were stratified into two age groups of 45–71 years and 72–87 years. The central EC density of corneas from 72 to 87 years donors was significantly lower (n = 18, 2423 ± 246 cells/mm^2^) than that of 45–71 years (n = 15; 2656 ± 209 cells/mm^2^; *p* = 0.0067, Figure 4B). However, there is no significant change between younger and older age groups in TZ width (45–71 years: 224 ± 52 µm; 72–87 years: 219 ± 35 µm) and TZ surface cell count (45–71 years: 2033 ± 282 cells/mm^2^; 72–87 years: 2175 ± 652 cells/mm^2^), as measured by HRTII/RCM (Figure 4C,D).

### 3.5. Ultrastructural Examination of the Endothelial Transition Zone

**Figure 5:**
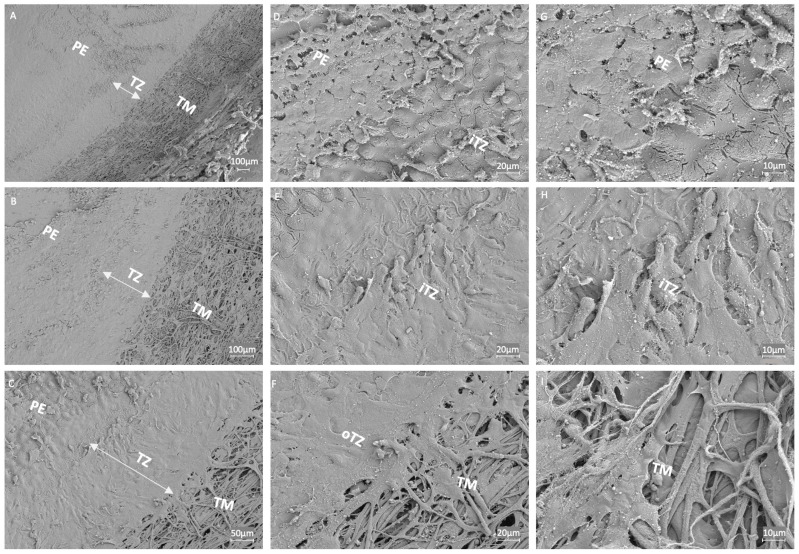
Scanning electron microscopy (SEM) images showing the ultrastructural organization of the inner transition zone (iTZ) and outer transition zone (oTZ), along with peripheral endothelium (PE), and trabecular meshwork (TM) regions. Panels (**A**–**C**) highlight the PE, TZ, and TM demarcation at 50×, 100×, and 200× magnifications in donor corneas. Panels (**E**,**H**) show microstructural changes in cellular morphology and extracellular matrix organization within TZ at 500× and 1000×. Visualization of ultrastructural organization variations between PE and inner TZ (**D**,**G**), and outer TZ and TM (**F**,**I**). Scale bars and magnifications are indicated within each image, with PE, TZ, and TM regions labeled for clarity.

Ultrastructural analysis of the corneal TZ and its adjacent regions, the PE and TM, was conducted using SEM at varying magnifications to assess tissue architecture and cellular organization. At lower magnifications (50×, 100×; Figure 5A,B), the TZ was distinctly identifiable as a smooth region, demarcating the rough-textured PE anteriorly and the fibrous-appearing TM posteriorly. At higher magnifications (200×; Figure 5C), anatomical distinctions between the inner TZ (iTZ) bordering the PE and the outer TZ (oTZ) adjacent to the TM became more apparent. A gradual increase in cellular density was visualized from the oTZ near the TM towards the iTZ adjoining the PE. Cells within the TZ displayed heterogeneity in size and polygonal morphology, compared to the more compact and uniformly arranged PE (Figure 5D–H). The cellular and extracellular components of the TZ were distinctly visible, suggesting active cell–matrix interactions (Figure 5D–F). Unlike the well-organized hexagonal or honeycomb-like lattice arrangement of the extracellular matrix (ECM) in Descemet’s membrane (DM), the ECM of the TZ lacked the s uniform organization (Figure S3). Within the TZ, cells were loosely arranged over a stromal matrix (Figure 5D,H). In contrast, PEs were compactly arranged in a tessellated fashion over the DM. The iTZ region exhibited irregularly distributed cells with interstitial spaces and matrix components, suggesting a potential structural basis for TZ’s dynamic role in cell migration and corneal endothelium regeneration, which requires further validation through functional studies. Meanwhile, in the oTZ, the cells appeared elongated and plate-like, bridging into the trabecular beams and the ECM of the TM (Figure 5F). TM showed increasingly fibrous and porous networks with large inter-fibrillary spaces (Figure 5I), providing evidence for pathways involved in fluid drainage.

To gain deeper insight into the ECM organization of the cornea, SEM was performed on the central stromal region beneath the peripheral endothelium (Figure S3). SEM images revealed long, thin, parallel collagen fibrils densely packed and arranged in flattened lamellae on top of one another. These parallel collagen fibrils were oriented at varying angles in adjacent lamellae, suggesting their role in providing mechanical strength and stability to the cornea. This ultrastructural collagen organization is also essential for maintaining corneal transparency [23].

### 3.6. Expression Analysis of Endothelial Transition Zone

**Figure 6:**
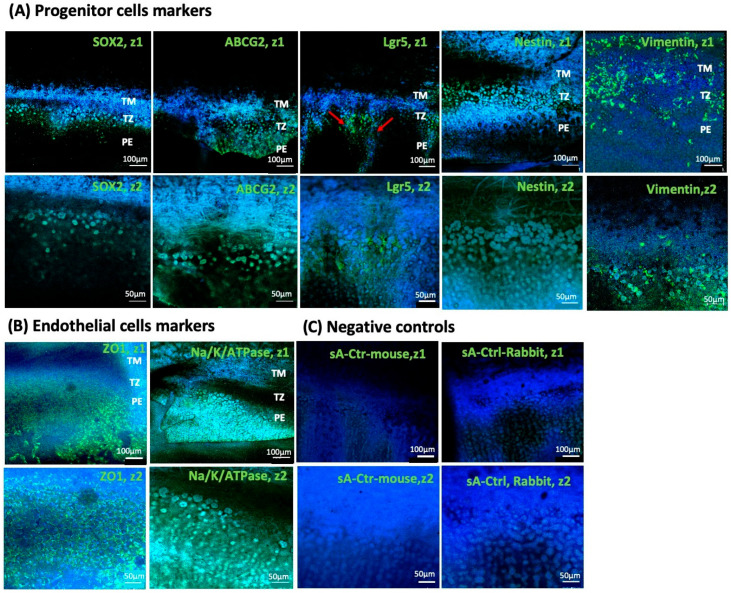
Representative immunohistochemistry images showing the expression of (**A**) progenitor/stem cells markers—SOX2, Nestin, Lgr5, ABCG2, Vimentin (in green), and (**B**) endothelial cells markers—ZO1 and Na/K-ATPase (in green) along with nuclear DAPI staining (in blue). Images were captured at 10× magnification: zoom 1 (z1) at 100 µm scale and zoom 2 (z2) at 50 µm scale. The top panel (z1) shows the broader anatomical region, including TM, TZ, and PE. Bottom panel (z2): magnified images highlighting cellular localization of (**A**) Sox2, ABCG2, Lgr5, Nestin, and Vimentin markers within the TZ, and (**B**) ZO1 and Na/K-ATPase in PE. (**C**) represents control staining with the corresponding secondary antibodies only. Examination and imaging were conducted with LSM780 (Carl Zeiss). TZ: Transition Zone, TM: Trabecular Meshwork, PE: Peripheral endothelium.

Immunohistochemical analysis detected the expression of stem cell markers Lgr5, ABCG2, Sox2, Vimentin, and Nestin in TZ cells, with distinct spatial expression patterns (Table 2). Lgr5 and Sox2 were predominantly expressed in the iTZ; ABCG2 expression was also higher in the iTZ, whereas Nestin was expressed throughout the entire TZ (Figure 6A). Additionally, we observed finger-like projections containing Lgr5-positive cell clusters extending from the inner TZ into the PE, supporting the hypothesis of progenitor cell migration.

The outer oTZ, which faces the TM, showed prominent Vimentin expression (Figure 6A), suggesting the mesenchymal characteristics of TZ cells and their potential involvement in TM regeneration. Notably, both Vimentin and Nestin were abundantly expressed in the TM. In contrast, their expression was comparatively lower in the PE, further suggesting a role for these intermediate filament proteins in TM remodeling processes. While endothelial markers like ZO1 and Na/K-ATPase were predominantly expressed mainly in PE, almost negligible expression of these endothelial markers was observed in TZ (Figure 6B, Table 2). A faint expression of Na/K-ATPase was observed in TM. These results indicate that the TZ may serve as a niche for progenitor cells.

## 4. Discussion

This study presents a comprehensive ex vivo analysis of the corneal TZ, a region that has received comparatively little attention in the evaluation of donor corneas. By combining phase-contrast microscopy, confocal imaging, ultrastructural, and immunohistochemical techniques, we have demonstrated that the TZ harbors a population of cells with stem/progenitor-like characteristics. The presence of these characteristics in the TZ is significant, given that standard methods of preserving donor corneas prior to transplantation result in a decrease in EC density, which is a critical parameter for assessing corneal health and donor tissue suitability. Interestingly, in our study, we observed a significant decline in EC density during storage time, while corneal TZ surface cell density remained largely unaffected. A decrease in EC density is often associated with age, trauma, surgery, corneal dystrophies, and glaucoma [1,24]. Our findings corroborate previous reports, showing a significant decrease in EC cell density with increasing age. However, no correlation was found between TZ cell density and age or gender.

To date, there has been no standardized method for measuring TZ width. In previous studies, TZ width was measured using SEM in donor corneas, but the results were variable [11,17], probably due to the experimental procedures, such as fixation or dehydration before imaging. Another method used was swept source optical coherence tomography (SS-OCT) to estimate TZ width, although the resolution does not allow direct visualization of TZ cell quantification [25]. In vivo confocal microscopy (HRT/RCM) is a confocal laser scanning microscope that allows high-resolution, in vivo examination of the cornea. It is a more cost-effective alternative to SEM, but it is currently no longer on the market. To date, in vivo confocal microscopy has been used to assess microstructures in various ocular tissues, including stromal cells, corneal sub-basal nerve plexus, corneal epithelium, and endothelium [20,26,27,28]. However, this is the first study to use ex vivo confocal microscopy (HRTII/RCM) to assess the structure of corneal TZ and its cell density in donor corneas. Furthermore, unlike SEM, this imaging technique does not require prior processing, thereby preserving the native tissue structure. The average TZ width in donor corneas from healthy individuals aged between 45 and 78 years was consistent with findings from recent studies on the endothelial TZ [17,29]. Although the TZ cell appeared irregular in shape and more sparsely arranged, with visible spaces in the TZ region as compared to densely packed endothelial cells, the average TZ cell density was only slightly lower than the central EC density. While Yam et al. reported a TZ cell density of approximately 700 cells/mm^2^ [17], this discrepancy could be due to differences in tissue preparation and imaging methodologies. In their study, the corneal rims were used, which involved trephination of the central corneal button, which may induce mechanical cell damage, possibly reducing TZ cell count. Furthermore, their TZ cell count procedure included fixation, permeabilization, and DAPI staining, which provided high accuracy in identifying nuclei but compromised native tissue integrity. In contrast, our ex vivo HRT/RCM imaging of donor corneas, including post-surgical tissue, allowed assessment of viable TZ cells without fixation, although the imaging may be affected by local architectural changes, and the optical resolution and depth penetration of HRT/RCM may lead to a miscalculation of the true cell density. However, DAPI staining could not be performed for imaging of the donor cornea with HRT/RCM due to its requirement for fixation, permeabilization, and its poor performance in labeling nuclei of viable cells. Another key difference is in the preservation of donor corneas. In the United States, corneas are stored at 4 °C in Optisol for up to 14 days, leading to progressive cell loss. In Europe, however, organ culture at 30–37 °C maintains metabolic activity for several weeks but may induce stress-related morphological changes. In our analysis, no significant age-related differences in TZ width and TZ cell density were observed between the two groups (45–71 years vs. 72–78 years), which aligns with the previous study [17]. This suggests that organ culture may be better for preserving TZ cells. However, most donor corneas in our cohort were from donors over 60 years of age (36 out of 41), with only five corneas from donors under 60 and limited representation of female donors (9 out of 41). As a result, potential gender-specific variation and age-related differences in young donors (<60 years) or longitudinal TZ cell density changes across broader age ranges cannot be excluded.

To further investigate the structural differences between corneal endothelium and TZ, unlike the tightly arranged endothelium, both HRTII/RCM and SEM revealed a loss of tight junctions between cells in the endothelial TZ, which was similar to previous studies in humans and animals [5,17]. SEM further revealed a disordered and loosely arranged extracellular matrix with interstitial spaces beneath the endothelial TZ cells, as observed by Yam et al. [17], which likely plays a role in maintaining stem cells in a quiescent state and preventing differentiation. By contrast, the DM and the stroma demonstrated a parallel, highly ordered dense fibrillary structure (Figure S3), which contributes to a more compact and stable organization of differentiated cells. The polygonal morphology of TZ cells indicates weaker attachment to the matrix compared to the hexagonal, flat cells of the endothelium. This loose attachment may facilitate the migration of cells to the PE or TM when necessary, promoting their differentiation.

The immunofluorescence analysis of the TZ with distinct expression patterns of stem cell markers SOX2, Nestin, Lgr5, Vimentin, and ABCG2 further suggests the presence of progenitor-like cells within this region. Lgr5 plays a critical role in maintaining the proliferative capacity of corneal endothelial progenitor cells through Wnt and Hedgehog signaling pathways [30]. SOX2 is a key transcription factor for maintaining pluripotency and directing the neural progenitor differentiation [31]. The increased expression of SOX2 and the appearance of endothelial markers in an embryonic stem-cell-based differentiation model [32] suggest its role in endothelial lineage commitment [33]. Consistent with previous findings [17,30], our study has reported prominent SOX2 and Lgr5 expression in the inner TZ of donor corneas, indicating the presence of neural progenitor-like cells with potential endothelial differentiation capacity. Similar to the study by Yam et al. [17], Lgr5 expression was extended into the PE from the inner TZ, with minimal expression in the outer TZ or TM, suggesting that Lgr5 may be primarily involved in the migration and proliferation of endothelial progenitor cells towards PE. These results align with previous findings in healthy corneas showing stem cell marker expression restricted to SL/TZ, but in wounded cornea, stem cell markers like SOX2 and PAX6 appeared in PE too, supporting the migration potential of TZ cells towards the injury site for endothelial repair [34]. Expression of ABCG2, Vimentin, and Nestin has previously been reported in SL/TZ region, TM, and PE [17,34,35], consistent with our findings. ABCG2, an ATP-binding cassette (ABC) transporter, provides cytoprotection against stress, toxins, and apoptosis [36,37,38], whereas Nestin and vimentin, intermediate filament proteins, are associated with cell migration and mesenchymal transition [39,40]. ABCG2 and Nestin are crucial for maintaining the undifferentiated state [39,41,42]. Vim-/- knockout mice have demonstrated a delay in cell migration at the wound healing site [43], indicating its critical role in promoting cell migration and tissue repair. In our study, the prominent expression of Nestin, Vimentin, and ABCG2 throughout the entire TZ, with a gradual decrease in expression observed in the TM and PE, suggests progenitors in an undifferentiated state of TZ cells, which may have migratory and proliferative potential in TM and PE regeneration. ZO1 and Na/K-ATPase are well-recognized markers of a fully differentiated endothelial phenotype. In our study, the absence of these markers in TZ cells indicates an undifferentiated state, which is consistent with the above-mentioned regenerative potential of these cells in maintaining corneal and TM homeostasis.

Given these findings, the TZ cells could differentiate into ECs to compensate for the cell loss in corneal dystrophies and in glaucoma. To date, only a few studies have demonstrated ex vivo expansion or differentiation of TZ cells in pig explants and in human models into endothelial-like cells expressing ZO1, Na/K-ATPase, and Col8A2 [17,44,45]. However, their therapeutic efficacy remains invalidated due to the absence of in vivo transplantation studies and functional assessment of the polarity, barrier, or ion transport activity. Moreover, corneal ECs often undergo mesenchymal transition during in vitro culture, losing critical functional properties such as barrier integrity, polarity, and pump functionality [46], which are critical for maintaining corneal hydration [44].

The possibility of in vivo/ex vivo migration of less differentiated cells, as evidenced by BrdU+/PAX6+/Sox2+ cells from the TZ or PE repopulating the EC and TM in human cornea, has been reported in normal physiological conditions as well as at the site of injury [4,34,47,48]. However, this has primarily been observed in corneas from younger donors (aged below 45), suggesting that age influences the proliferative and migratory capacity of these cells. This could also be attributed to an increase in TGF beta expression or other cell cycle inhibitors in aqueous humor with age [49], which could inhibit their differentiation in EC. In our study, progenitor cell density remained constant during aging or storage conditions; however, we did not quantify the stem cell population in donor corneas for age, as most of the corneas used for immunostaining were from older donors. Interestingly, repopulation of cells at injury sites, probably migrated from the SL/TZ after laser trabeculotomy, was observed both in glaucoma patients (aged 43 to 83 years) and in organ culture models, suggesting that these cells respond to injury stimuli regardless of age [16,50]. Administration of ROCK inhibitors (Y-27632), a TGF-beta inhibitor, and growth factors (Cdk4, Ccnd1, Myc, Sox2, Yap, and EGF, FGF, PDGF, NGF) has demonstrated efficient corneal endothelial repopulation in vivo as well as in vitro in aging, injury, and disease conditions [17,44,51,52,53,54]. Thus, rather than relying solely on ex vivo expansion of TZ cells in culture—which can introduce morphological and functional variability, technical challenges, high costs, and time consuming procedures—stimulating endogenous TZ-derived cells within donor or patient corneas via topical or injectable delivery of growth factors (like EGF, FGF, PDGF) or ROCK/TGFβ inhibitors in vivo or supplementing donor corneas with these factors ex vivo may have potential for more effective, long-term, and durable regenerative outcomes. For such strategies, in vivo imaging techniques like HRT/RCM can be instrumental in identifying, monitoring, and targeting TZ cells and evaluating pre- and post-treatment counts.

A limitation of this study is that, in accordance with German transplantation law and data protection regulations, only donor information directly relevant to tissue safety and suitability was available (e.g., age, diabetes status, lens status). A detailed systemic medication history or cause of death could not be obtained, which may influence EC or TZ count and, therefore, the interpretation of donor tissue quality.

Furthermore, information on TZ cell counts or TZ width in specific corneal quadrants was unavailable, as the corneas were not routinely marked during retrieval. Therefore, regional TZ cell distribution could not be assessed. Yam et al. reported the smallest TZ width in the nasal quadrant [17], suggesting quadrant-specific differences. Future studies involving marked corneas are required to confirm these regional variations.

We would like to acknowledge that this study represents a first step towards understanding the cellular and structural features of the TZ. While our findings offer new insights into the architecture and the potential regenerative capacity of TZ, further translational studies are required to evaluate the relevance for donor cornea assessment and transplantation outcomes. TZ quantification revealed inter-donor variability, but the biological and functional relevance of these differences requires further validation. In addition, a more comprehensive characterization of the progenitor-like cells and their possible contribution to tissue regeneration of the central corneal endothelium is needed. Overall, our results provide a promising foundation for future research aiming to leverage the TZ for improved donor tissue evaluation and to potentially expand the usable donor pool.

In conclusion, our multimodal imaging approach—including reflectance confocal microscopy—enabled detailed characterization of the corneal transition zone (TZ) as a niche enriched with stem/progenitor-like cells expressing SOX2, Lgr5, ABCG2, Vimentin, and Nestin. SEM revealed distinct TZ micro-architecture with polygonal cells and dynamic ECM interactions indicative of regenerative potential. Reflectance confocal microscopy (HRTII/RCM) allowed non-invasive, high-resolution visualization of TZ cell dynamics ex vivo. This method offers a practical tool for improving donor tissue assessment by incorporating TZ-specific parameters. Harnessing the regenerative potential of TZ may help reduce donor cornea discard rates and support future cell-based therapies.

## Figures and Tables

**Table 1:** Characteristics of the donor cornea and the cell density parameter.

Donor Corneas (n = 41)	Minimum	Maximum	Mean ± SD
Age (years)	45	87	71.56 ± 10.90
Storage time (days)	15	35	23.39 ± 5.72
EC count (per mm^2^)	1110	2980	2382.80 ± 405.64
TZ cell count (per mm^2^)	925	2975	2107.81 ± 505.03
TZ width (µm)	142.25	299.00	221.11 ± 40.93

EC: endothelial cells, TZ: transition zone, SD: standard deviation.

**Table 2:** Expression of stem/progenitor and corneal endothelial markers in trabecular meshwork (TM), transition zone (TZ), and peripheral endothelium (PE).

Markers	No. of Sample	Expression		
		TM	TZ	PE
ZO1	4	−	−	+++
Na/K-ATPase	4	+	−	+++
Lgr5	4	−	++	++
ABCG2	3	+	+++	++
SOX2	3	−	+++	−
Nestin	4	++	+++	+
Vimentin	4	++	+++	+

+++ denotes strong, ++ denotes moderate, + denotes weak, − denotes absent.

## Data Availability

The data that support the findings of this study are available from the corresponding author upon reasonable request.

## Supplementary Materials

The following supporting information can be downloaded at: https://www.mdpi.com/article/10.3390/cells14231851/s1, Figure S1: Representative Phase-contrast microscopy images of central corneal endothelial cells in a donor cornea, showing the characteristic hexagonal, tessellated arrangement at different magnifications: (A) 10×, (B) 20×, and (C) 32×; Figure S2: Effect of age on endothelial cell density in male and female donor corneas; Figure S3: Scanning electron microscopy (SEM) images showing the ultrastructural organization of the corneal stroma in donor corneas; Table S1: Association of age, endothelial cell count, TZ width and TZ cell count of donor corneas with gender; Table S2: List of primary and secondary antibodies used for immunostaining of endothelial TZ, TM and PE in donor cornea.

## Abbreviations

The following abbreviations are used in this manuscript:

TZ	Transition Zone
iTZ	Inner Transition Zone
oTZ	Outer Transition Zone
EC	Endothelial Cell
PE	Peripheral Endothelium
TM	Trabecular Meshwork
HRTII/RCM	Heidelberg Retina Tomograph equipped with Rostock Cornea Module
SEM	Scanning Electron Microscopy
TGF-β	Transforming Growth Factor-Beta
FECD	Fuchs’endothelial corneal dystrophy
DM	Descemet’s membrane
SL	Schwalbe’s line
RT	Room temperature
CTCF	Corrected total cell fluorescence
ECM	Extracellular matrix
SD	Standard deviation
DGFG	German Society for Tissue Transplantation

## References

[1] Vaiciuliene R., Rylskyte N., Baguzyte G., Jasinskas V. (2022). Risk factors for fluctuations in corneal endothelial cell density (review). Exp. Ther. Med..

[2] Schroeter J., Rieck P. (2009). Endothelial evaluation in the cornea bank. Dev. Ophthalmol..

[3] Bu J.B., Grabitz S.D., Schon F., Apel M., Pusch T., Gericke A., Poplawski A., Pfeiffer N., Wasielica-Poslednik J. (2024). Utility of a Model to Predict Endothelial Cell Density of Donor Corneas to Determine Suitability for Transplantation. Transl. Vis. Sci. Technol..

[4] Whikehart D.R., Parikh C.H., Vaughn A.V., Mishler K., Edelhauser H.F. (2005). Evidence suggesting the existence of stem cells for the human corneal endothelium. Mol. Vis..

[5] Lee J.S., Lee S.Y., Chin H.S., Kim N.R., Jung J.W. (2024). Microstructure of the corneal endothelial transition zone in different laboratory animals. Mol. Vis..

[6] Eghrari A.O., Riazuddin S.A., Gottsch J.D. (2015). Overview of the Cornea: Structure, Function, and Development. Prog. Mol. Biol. Transl. Sci..

[7] Meek K.M., Knupp C. (2015). Corneal structure and transparency. Prog. Retin. Eye Res..

[8] Joyce N.C., Harris D.L., Mello D.M. (2002). Mechanisms of mitotic inhibition in corneal endothelium: Contact inhibition and TGF-β2. Investig. Ophthalmol. Vis. Sci..

[9] Paull A.C., Whikehart D.R. (2005). Expression of the p53 family of proteins in central and peripheral human corneal endothelial cells. Mol. Vis..

[10] Xiao Y., McGhee C.N.J., Zhang J. (2024). Adult stem cells in the eye: Identification, characterisation, and therapeutic application in ocular regeneration—A review. Clin. Exp. Ophthalmol..

[11] Breazzano M.P., Fikhman M., Abraham J.L., Barker-Griffith A.E. (2013). Analysis of Schwalbe’s Line (Limbal Smooth Zone) by Scanning Electron Microscopy and Optical Coherence Tomography in Human Eye Bank Eyes. J. Ophthalmic Vis. Res..

[12] Katikireddy K.R., Schmedt T., Price M.O., Price F.W., Jurkunas U.V. (2016). Existence of Neural Crest-Derived Progenitor Cells in Normal and Fuchs Endothelial Dystrophy Corneal Endothelium. Am. J. Pathol..

[13] Tripathi B.J., Tripathi R.C. (1989). Neural crest origin of human trabecular meshwork and its implications for the pathogenesis of glaucoma. Am. J. Ophthalmol..

[14] Balachandran C., Ham L., Verschoor C.A., Ong T.S., van der Wees J., Melles G.R. (2009). Spontaneous corneal clearance despite graft detachment in descemet membrane endothelial keratoplasty. Am. J. Ophthalmol..

[15] Arbelaez J.G., Price M.O., Price F.W. (2014). Long-term follow-up and complications of stripping descemet membrane without placement of graft in eyes with Fuchs endothelial dystrophy. Cornea.

[16] Alexander R.A., Grierson I. (1989). Morphological effects of argon laser trabeculoplasty upon the glaucomatous human meshwork. Eye.

[17] Yam G.H., Seah X., Yusoff N., Setiawan M., Wahlig S., Htoon H.M., Peh G.S.L., Kocaba V., Mehta J.S. (2019). Characterization of Human Transition Zone Reveals a Putative Progenitor-Enriched Niche of Corneal Endothelium. Cells.

[18] Braunger B.M., Ademoglu B., Koschade S.E., Fuchshofer R., Gabelt B.T., Kiland J.A., Hennes-Beann E.A., Brunner K.G., Kaufman P.L., Tamm E.R. (2014). Identification of adult stem cells in Schwalbe’s line region of the primate eye. Investig. Ophthalmol. Vis. Sci..

[19] Du Y., Roh D.S., Mann M.M., Funderburgh M.L., Funderburgh J.L., Schuman J.S. (2012). Multipotent stem cells from trabecular meshwork become phagocytic TM cells. Investig. Ophthalmol. Vis. Sci..

[20] Stachs O., Guthoff R.F., Aumann S., Bille J.F. (2019). In Vivo Confocal Scanning Laser Microscopy. High Resolution Imaging in Microscopy and Ophthalmology: New Frontiers in Biomedical Optics.

[21] Eckard A., Stave J., Guthoff R.F. (2006). In vivo investigations of the corneal epithelium with the confocal Rostock Laser Scanning Microscope (RLSM). Cornea.

[22] Stachs O., Zhivov A., Kraak R., Stave J., Guthoff R. (2007). In vivo three-dimensional confocal laser scanning microscopy of the epithelial nerve structure in the human cornea. Graefe’s Arch. Clin. Exp. Ophthalmol..

[23] Espana E.M., Birk D.E. (2020). Composition, structure and function of the corneal stroma. Exp. Eye Res..

[24] Islam Q.U., Saeed M.K., Mehboob M.A. (2017). Age related changes in corneal morphological characteristics of healthy Pakistani eyes. Saudi J. Ophthalmol..

[25] Wahlig S., Yam G.H., Chong W., Seah X.Y., Kocaba V., Ang M., Htoon H.M., Tun T.A., Ong H.S., Mehta J.S. (2018). Quantification of the Posterior Cornea Using Swept Source Optical Coherence Tomography. Transl. Vis. Sci. Technol..

[26] Misra S.L., Goh Y.W., Patel D.V., Riley A.F., McGhee C.N. (2015). Corneal microstructural changes in nerve fiber, endothelial and epithelial density after cataract surgery in patients with diabetes mellitus. Cornea.

[27] Kohler B., Allgeier S., Bartschat A., Guthoff R.F., Bohn S., Reichert K.M., Stachs O., Winter K., Mikut R. (2017). In vivo imaging of the corneal nerve plexus: From single image to large scale map. Ophthalmologe.

[28] McCarey B.E., Edelhauser H.F., Lynn M.J. (2008). Review of corneal endothelial specular microscopy for FDA clinical trials of refractive procedures, surgical devices, and new intraocular drugs and solutions. Cornea.

[29] Jonas J.B., Panda-Jonas S., Mehta J.S., Jonas R.A. (2025). Anatomic Relationship Among Descemet’s Membrane, Trabecular Meshwork, Scleral Spur, and Ciliary Muscle. Investig. Ophthalmol. Vis. Sci..

[30] Hirata-Tominaga K., Nakamura T., Okumura N., Kawasaki S., Kay E.P., Barrandon Y., Koizumi N., Kinoshita S. (2013). Corneal endothelial cell fate is maintained by LGR5 through the regulation of hedgehog and Wnt pathway. Stem Cells.

[31] Zhang S., Cui W. (2014). Sox2, a key factor in the regulation of pluripotency and neural differentiation. World J. Stem Cells.

[32] Yao Y., Yao J., Bostrom K.I. (2019). SOX Transcription Factors in Endothelial Differentiation and Endothelial-Mesenchymal Transitions. Front. Cardiovasc. Med..

[33] Zhu Y.T., Li F., Han B., Tighe S., Zhang S., Chen S.Y., Liu X., Tseng S.C. (2014). Activation of RhoA-ROCK-BMP signaling reprograms adult human corneal endothelial cells. J. Cell Biol..

[34] McGowan S.L., Edelhauser H.F., Pfister R.R., Whikehart D.R. (2007). Stem cell markers in the human posterior limbus and corneal endothelium of unwounded and wounded corneas. Mol. Vis..

[35] Yun H., Zhou Y., Wills A., Du Y. (2016). Stem Cells in the Trabecular Meshwork for Regulating Intraocular Pressure. J. Ocul. Pharmacol. Ther..

[36] Krishnamurthy P., Ross D.D., Nakanishi T., Bailey-Dell K., Zhou S., Mercer K.E., Sarkadi B., Sorrentino B.P., Schuetz J.D. (2004). The stem cell marker Bcrp/ABCG2 enhances hypoxic cell survival through interactions with heme. J. Biol. Chem..

[37] Zhou S., Morris J.J., Barnes Y., Lan L., Schuetz J.D., Sorrentino B.P. (2002). *Bcrp1* gene expression is required for normal numbers of side population stem cells in mice, and confers relative protection to mitoxantrone in hematopoietic cells in vivo. Proc. Natl. Acad. Sci. USA.

[38] Kubota M., Shimmura S., Miyashita H., Kawashima M., Kawakita T., Tsubota K. (2010). The anti-oxidative role of ABCG2 in corneal epithelial cells. Investig. Ophthalmol. Vis. Sci..

[39] Tong Z., Yin Z. (2024). Distribution, contribution and regulation of nestin^+^ cells. J. Adv. Res..

[40] Sivagurunathan S., Vahabikashi A., Yang H., Zhang J., Vazquez K., Rajasundaram D., Politanska Y., Abdala-Valencia H., Notbohm J., Guo M. (2022). Expression of vimentin alters cell mechanics, cell-cell adhesion, and gene expression profiles suggesting the induction of a hybrid EMT in human mammary epithelial cells. Front. Cell Dev. Biol..

[41] Park D., Xiang A.P., Mao F.F., Zhang L., Di C.G., Liu X.M., Shao Y., Ma B.F., Lee J.H., Ha K.S. (2010). Nestin is required for the proper self-renewal of neural stem cells. Stem Cells.

[42] Scharenberg C.W., Harkey M.A., Torok-Storb B. (2002). The *ABCG2* transporter is an efficient Hoechst 33342 efflux pump and is preferentially expressed by immature human hematopoietic progenitors. Blood.

[43] Eckes B., Colucci-Guyon E., Smola H., Nodder S., Babinet C., Krieg T., Martin P. (2000). Impaired wound healing in embryonic and adult mice lacking vimentin. J. Cell Sci..

[44] Zhang J., Ahmad A.M., Ng H., Shi J., McGhee C.N.J., Patel D.V. (2020). Successful culture of human transition zone cells. Clin. Exp. Ophthalmol..

[45] Yam G.H., Pi S., Du Y., Mehta J.S. (2023). Posterior corneoscleral limbus: Architecture, stem cells, and clinical implications. Prog. Retin. Eye Res..

[46] Roy O., Leclerc V.B., Bourget J.M., Theriault M., Proulx S. (2015). Understanding the process of corneal endothelial morphological change in vitro. Investig. Ophthalmol. Vis. Sci..

[47] Schimmelpfennig B.H. (1984). Direct and indirect determination of nonuniform cell density distribution in human corneal endothelium. Investig. Ophthalmol. Vis. Sci..

[48] Daus W., Volcker H.E., Meysen H., Bundschuh W. (1989). Vital staining of the corneal endothelium--increased possibilities of diagnosis. Fortschr. Ophthalmol..

[49] Trivedi R.H., Nutaitis M., Vroman D., Crosson C.E. (2011). Influence of race and age on aqueous humor levels of transforming growth factor-beta 2 in glaucomatous and nonglaucomatous eyes. J. Ocul. Pharmacol. Ther..

[50] Acott T.S., Samples J.R., Bradley J.M., Bacon D.R., Bylsma S.S., Van Buskirk E.M. (1989). Trabecular repopulation by anterior trabecular meshwork cells after laser trabeculoplasty. Am. J. Ophthalmol..

[51] Okumura N., Koizumi N., Kay E.P., Ueno M., Sakamoto Y., Nakamura S., Hamuro J., Kinoshita S. (2013). The ROCK inhibitor eye drop accelerates corneal endothelium wound healing. Investig. Ophthalmol. Vis. Sci..

[52] Okumura N., Kay E.P., Nakahara M., Hamuro J., Kinoshita S., Koizumi N. (2013). Inhibition of TGF-β signaling enables human corneal endothelial cell expansion in vitro for use in regenerative medicine. PLoS ONE.

[53] Eveleth D., Pizzuto S., Weant J., Jenkins-Eveleth J., Bradshaw R.A. (2020). Proliferation of Human Corneal Endothelia in Organ Culture Stimulated by Wounding and the Engineered Human Fibroblast Growth Factor 1 Derivative TTHX1114. J. Ocul. Pharmacol. Ther..

[54] Rompolas P., Curtis E., Ramarapu R., Marshall M., Yoo H., Park S., Leonard B., Thomasy S.M. (2025). Unlocking the therapeutic potential of the corneal endothelium: Intravital imaging reveals endogenous regenerative capabilities. Investig. Ophthalmol. Vis. Sci..

