# Supplementary material for: Multimodal Imaging of the Corneal Endothelial Transition Zone Reveals Progenitor Cell Population
Source: Cells. 2025 Nov 25;14(23):1851. doi: 10.3390/cells14231851 (PMC12691442; doi:10.3390/cells14231851)
Supplement: Supplementary file 1 [file cells-14-01851-s001.zip › cells-3920680-supplementary.pdf]

Supplementary Material:

Table S1: Association of age, endothelial cell count, TZ width, and cell count of donor corneas with gender.

Gender	Male	Female	p-value
Number of samples	32	9	--
Age (years)	71.2 ± 10.6	73 ± 12.4	0.6667
Endothelial cell count (cells/mm²)	2362.7 ± 424,89	2454.4 ± 340.26	0.5601
TZ width (µm)	221.2 ± 37.4	220.95 ± 54.38	0.9873
TZ cell count (cells/mm²)	2107.8 ± 505.03	2319.4 ± 506.02	0.2738

Table S2: List of primary and secondary antibodies used for immunostaining of endothelial TZ, TM, and PE in donor cornea

Markers/ Antibodies	Host	Catalogue number	Concentration (in PBS)
Vimentin	Mouse	sc-32322 (Santa Cruz)	1:50
Nestin	Mouse	sc-23927 (Santa Cruz)	1:50
ABCG2	Mouse	sc-377176(Santa Cruz)	1:50
Lgr5	Mouse	TA503316 (OriGene)	1:50
Sox2	Mouse	sc-365823 (Santa Cruz)	1:50
NaK-ATPase	Mouse	ab7671 (abcam)	1:50
ZO1	Rabbit	40-2200 (Invitrogen)	1:25
Anti-Mouse Alexa Fluor488	Donkey	715-545-151 (dianova)	1:100
Anti-Rabbit Alexa Fluor488	Donkey	715-545-152 (dianova)	1:50

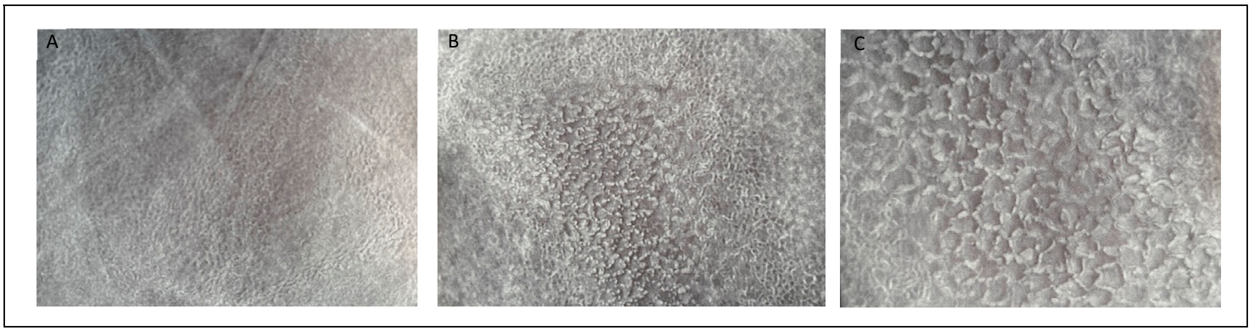

Figure S1: Representative Phase-contrast microscopy images of central corneal endothelial cells in a donor cornea, showing the characteristic hexagonal, tessellated arrangement at different magnifications: (A) 10×, (B) 20×, and (C) 32×.

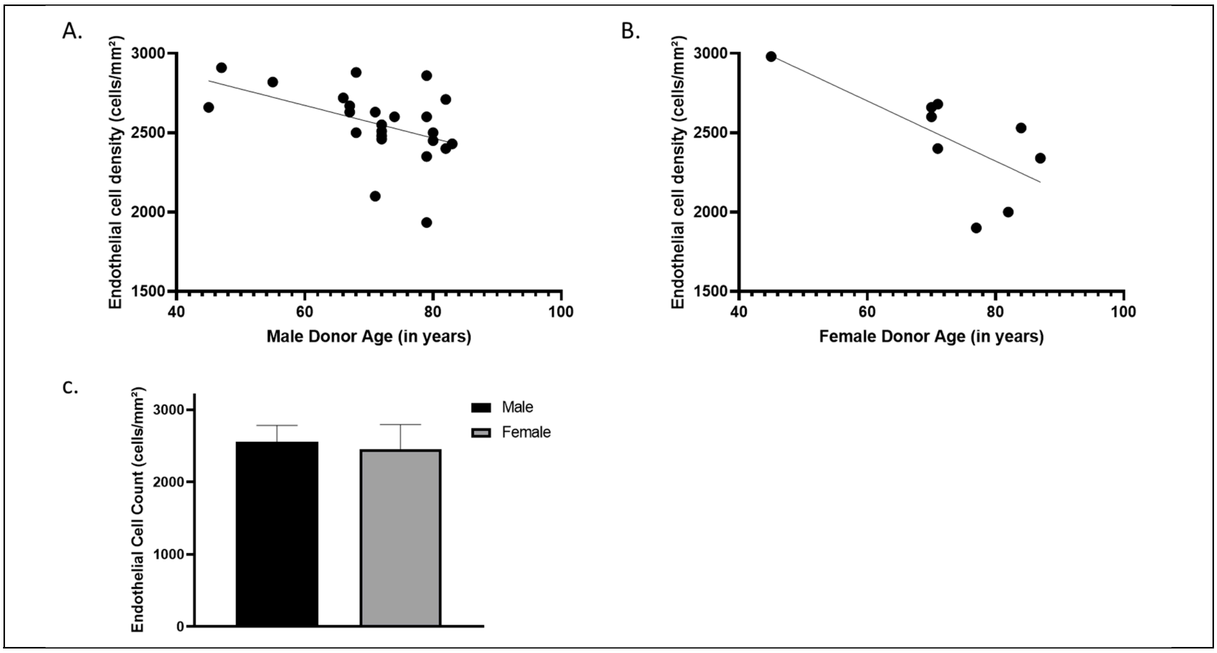

Figure S2: Effect of age on endothelial cell density in male and female donor corneas. male donor cornea (n = 32), female donor cornea (n = 9).

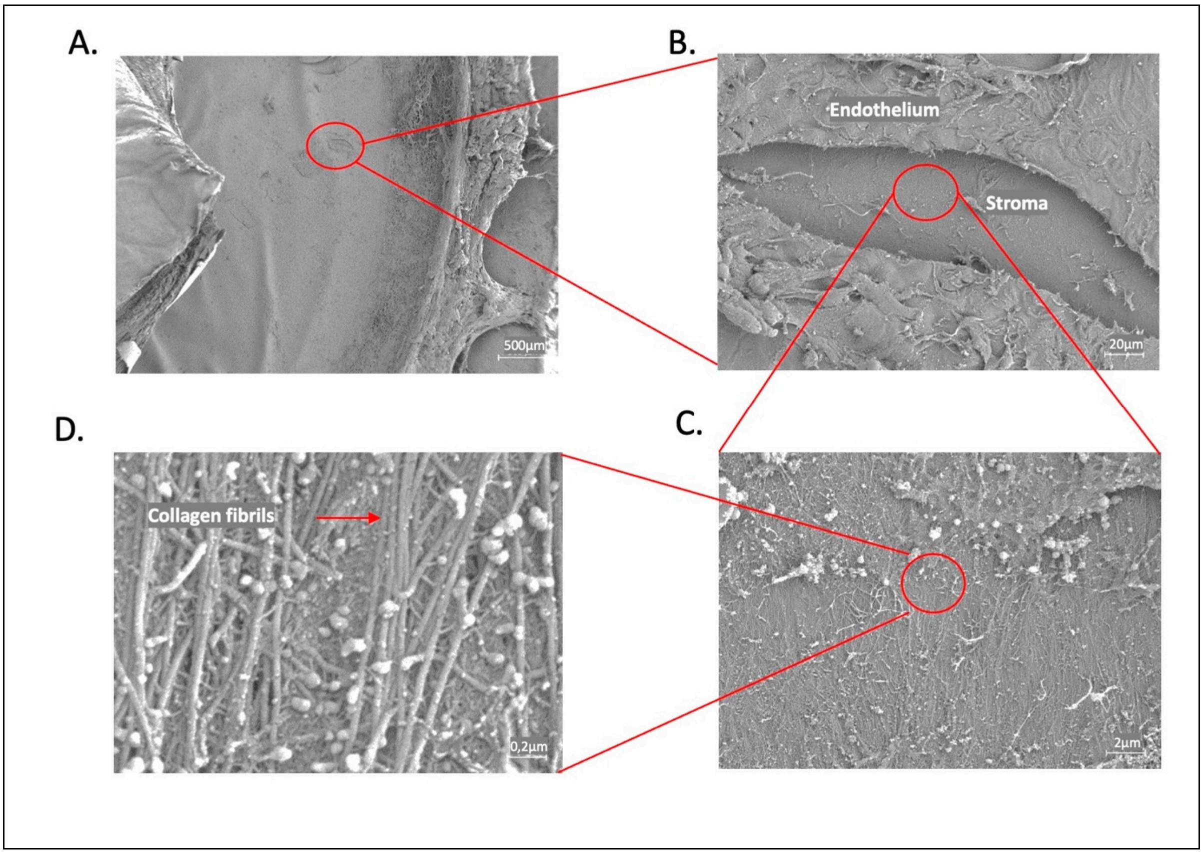

Figure S3: Scanning electron microscopy (SEM) images showing the ultrastructural organization of the corneal stroma in donor corneas. Panel (A) with the red circle highlighting the stroma region magnified in Panel (B) at 500× image, revealing the stroma as a fibrous network beneath the endothelial layer (top layer). Panel (C) at 5,000× displaying individual collagen fibrils within the stroma. Panel (D) at magnification 50,000× provides ultrastructural details of collagen fibrils. (FE-SEM Merlin compact, Carl Zeiss).
